# The role of synovial macrophages and macrophage-produced cytokines in driving aggrecanases, matrix metalloproteinases, and other destructive and inflammatory responses in osteoarthritis

**DOI:** 10.1186/ar2099

**Published:** 2006-12-19

**Authors:** Jan Bondeson, Shane D Wainwright, Sarah Lauder, Nick Amos, Clare E Hughes

**Affiliations:** 1 Department of Rheumatology, Cardiff University, Heath Park, Cardiff, CF14 4XN, UK; 2 Connective Tissue Biology Laboratories, Cardiff School of Biosciences, Cardiff University, Museum Avenue, Cardiff, CF10 3US, UK

## Abstract

There is an increasing body of evidence that synovitis plays a role in the progression of osteoarthritis and that overproduction of cytokines and growth factors from the inflamed synovium can influence the production of degradative enzymes and the destruction of cartilage. In this study, we investigate the role of synovial macrophages and their main proinflammatory cytokines, interleukin (IL)-1 and tumour necrosis factor-alpha (TNF-α), in driving osteoarthritis synovitis and influencing the production of other pro- and anti-inflammatory cytokines, production of matrix metalloproteinases, and expression of aggrecanases in the osteoarthritis synovium. We established a model of cultures of synovial cells from digested osteoarthritis synovium derived from patients undergoing knee or hip arthroplasties. By means of anti-CD14-conjugated magnetic beads, specific depletion of osteoarthritis synovial macrophages from these cultures could be achieved. The CD14^+^-depleted cultures no longer produced significant amounts of macrophage-derived cytokines like IL-1 and TNF-α. Interestingly, there was also significant downregulation of several cytokines, such as IL-6 and IL-8 (*p *< 0.001) and matrix metalloproteinases 1 and 3 (*p *< 0.01), produced chiefly by synovial fibroblasts. To investigate the mechanisms involved, we went on to use specific downregulation of IL-1 and/or TNF-α in these osteoarthritis cultures of synovial cells. The results indicated that neutralisation of both IL-1 and TNF-α was needed to achieve a degree of cytokine (IL-6, IL-8, and monocyte chemoattractant protein-1) and matrix metalloproteinase (1, 3, 9, and 13) inhibition, as assessed by enzyme-linked immunosorbent assay and by reverse transcription-polymerase chain reaction (RT-PCR), similar to that observed in CD14^+^-depleted cultures. Another interesting observation was that in these osteoarthritis cultures of synovial cells, IL-1β production was independent of TNF-α, in contrast to the situation in rheumatoid arthritis. Using RT-PCR, we also demonstrated that whereas the ADAMTS4 (a disintegrin and metalloprotease with thrombospondin motifs 4) aggrecanase was driven mainly by TNF-α, ADAMTS5 was not affected by neutralisation of IL-1 and/or TNF-α. These results suggest that, in the osteoarthritis synovium, both inflammatory and destructive responses are dependent largely on macrophages and that these effects are cytokine-driven through a combination of IL-1 and TNF-α.

## Introduction

Osteoarthritis (OA), one of the most common diseases among mammals, can be considered as part of the ageing process. Mechanical factors such as a history of joint trauma or a high body mass index are recognised risk factors for OA, as are certain endogenous factors like type II collagen mutations and acetabular dysplasia. There is also a growing body of evidence that synovial inflammation is implicated in many of the signs and symptoms of OA, including joint swelling and effusion [1,2]. Histologically, the OA synovium shows hyperplasia with an increased number of lining cells and a mixed inflammatory infiltrate consisting mainly of macrophages [3]. Some degree of synovitis has been reported even in early OA [2].

Synovitis in OA is likely to contribute to disease progression, as judged by the correlation between biological markers of inflammation, like C-reactive protein and cartilage oligomeric matrix protein, with the progression of structural changes in OA [4-6]. The overproduction of cytokines and growth factors from the inflamed synovium may play a role in the pathophysiology of OA. The low-grade OA synovitis is itself cytokine-driven, although the levels of proinflammatory cytokines are lower than in rheumatoid arthritis (RA). In particular, tumour necrosis factor-alpha (TNF-α) and interleukin (IL)-1 have been suggested as key players in OA pathogenesis [7-9], both in synovial inflammation and in activation of chondrocytes. These cytokines can stimulate their own production and induce synovial cells and chondrocytes to produce IL-6, IL-8, and leukocyte inhibitory factor as well as stimulate protease and prostaglandin production [1,10]. The hypothesis that TNF-α and IL-1 are key mediators of articular cartilage destruction has raised the possibility of anti-cytokine therapy in OA or the design of disease-modifying osteoarthritic drugs [1,11,12].

If it is accepted that synovial inflammation and the production of proinflammatory and destructive mediators from the OA synovium are of importance for the symptoms and progression of OA, it is an important question which cell type in the OA synovium is responsible for maintaining synovial inflammation. In RA, in which the macrophage is the main promoter of disease activity, macrophage-produced TNF-α is a major therapeutic target. Unfortunately, much less is known about the role of macrophages in OA. Histological studies have demonstrated that OA synovial macrophages exhibit an activated phenotype and that they produce both proinflammatory cytokines and vascular endothelial growth factor [13]. Synovial macrophage differentiation differs between inflammatory and non-inflammatory OA [14]. In mouse models of osteophyte formation induced by injections of transforming growth factor-beta or of collagenase, depletion of macrophages by injection of clodronate-laden liposomes led to marked inhibition of osteophytes, suggesting that these cells are important for this form of structural damage [15,16].

Some of the matrix metalloproteinases (MMPs) have degradative effects on the extracellular matrix and have been suggested [17,18] as important cofactors or disease mediators in OA. MMP-1 and MMP-13 are capable of cleaving collagen type II, whereas MMP-3 is active against other components of the extracellular matrix, such as fibronectin and laminin. Although there has been some interest in MMP inhibitors as therapeutic agents in OA [19-21], the importance of these molecular pathways is not entirely clear, nor are there conclusive data on whether macrophages and macrophage-produced cytokines stimulate MMP production in the OA synovium.

Articular cartilage contains high concentrations of the large aggregating proteoglycan aggrecan. The high negative charge density of the glycosaminoglycan chains on aggrecan monomers in cartilage proteoglycan is essential for the ability of articular cartilage to withstand compressive deformation. The depletion of aggrecan from articular cartilage, as evidenced by the release of aggrecan fragments into the synovial fluid, is a central pathophysiological event in OA. It has been demonstrated that the release of aggrecan from both normal and OA cartilage involves a specific cleavage by a group of enzymes known as the aggrecanases and that it does not involve the MMPs [22,23]. The aggrecanases are members of the family of the ADAMTS (a disintegrin and metalloprotease with thrombospondin motifs). To date, several such enzymes have been identified, among them aggrecanase-1 (ADAMTS4) [24] and aggrecanase-2 (ADAMTS5) [25]. IL-1 and TNF-α can mediate the catabolism of aggrecan, but whereas ADAMTS5 is constitutively expressed, ADAMTS4 is induced after IL-1 or TNF treatment of cartilage explants [26]. It has not been ascertained whether these cytokines act directly on the aggrecanase enzyme; however, in OA cartilage, aggrecanase is upregulated in the absence of catabolic stimulation [27]. More information is needed about the regulation of the aggrecanases, in particular whether they are driven by proinflammatory cytokines produced by macrophages or other cells.

We set up a model of cultures of synovial cells from digested OA synovium. These cells have the advantage of spontaneously producing a variety of both pro- and anti-inflammatory cytokines, including TNF-α, IL-1, and IL-10, as well as the major MMPs and tissue inhibitors of MMPs (TIMPs) [28]. In this study, we used anti-CD14-conjugated magnetic beads to achieve specific depletion of OA synovial macrophages in order to investigate the importance of these macrophages for the spontaneous production of proinflammatory cytokines and destructive MMPs in the OA synovium. We also assessed, by means of specific neutralisation of macrophage-produced TNF-α and IL-1, the contribution of these two proinflammatory cytokines on the production of each other, on other proinflammatory mediators, on the expression and production of various MMPs and TIMPs, and on ADAMTS4 and ADAMTS5 gene expression.

## Materials and methods

### Preparation of synovial specimens

OA synovial specimens were obtained from 19 patients undergoing knee or hip replacement surgery (14 females, 5 males; ages 55 to 84 years) after ethical permission had been given by the Gwent and Bro Taf National Health Service Trusts ethical committees. None of these patients was taking corticosteroids or any kind of disease-modifying antirheumatic drug. The synovium was dissociated by cutting it into small pieces and digesting it with collagenase and DNAse as described [28]. After the 2-hour digestion, the cell suspension was filtered through a 100-μm filter (BD Biosciences, San Jose, CA) to remove any undigested tissue. The filtered cell suspension was centrifuged at 300 *g *for 10 minutes. The supernatant was collected and centrifuged again for 10 minutes at 300 *g*. After the second spin, the two pellets were combined and resuspended into 10 ml of RPMI medium 1640 (with 2 mM l-glutamine and 10 μg/ml penicillin-streptomycin) supplemented with 10% heat-inactivated foetal calf serum (FCS). Flow cytometry analysis of the OA synovial cells showed that the synovium was comprised mainly of fibroblast-like synoviocytes with 2% to 7% macrophages, less than 0.5% neutrophils, and less than 0.1% T cells.

### Peripheral blood mononuclear cell preparation

The macrophage depletion system was tested using peripheral blood mononuclear cells (PBMCs) to ensure that the depletion would be effective. Whole blood was extracted from healthy human volunteers and separated out using Ficoll-Hypaque (Sigma-Aldrich, St. Louis, MO, USA) density gradient centrifugation. The PBMCs were taken off, washed, counted, and resuspended in 10 ml of RPMI 1640 medium (supplemented as above).

### Macrophage depletion

A magnetic-activated cell sorting (MACS) separation column system (Miltenyi Biotec, Bergisch Gladbach, Germany) was assembled by placing a MiniMacs separation column into the magnetic component and was washed with 500 μl of depletion buffer. To prepare a population of cells depleted of CD14^+ ^cells, either PBMCs or synovial cells were first passed through a 30-μm MiniMacs filter to remove cell aggregates. The resulting cell suspension was centrifuged at 300 *g *for 10 minutes. The pellet was resuspended into 80 μl of depletion buffer (comprising phosphate-buffered saline [pH 7.2] supplemented with 0.5% bovine serum albumin and 2 mM EDTA) per 10^7 ^cells. Half the cells were removed to act as the total (undepleted) cell population. Twenty microlitres of MACS CD14 microbeads was added per 10^7 ^cells to the other half, which was then mixed thoroughly and incubated on ice for 15 minutes before being washed and resuspended in 500 μl of depletion buffer.

The CD14 magnetically labelled cells were applied to the column and the effluent was collected. Then, 3 × 500 μl of depletion buffer was applied to the column and the effluent was collected. The effluent (2 ml) contained the cells depleted of CD14^+ ^cells, as the magnetically labelled CD14-labelled cells were retained within the separation column.

To deplete T cells from the synovial cell culture, 20 μl of CD3 microbeads was added per 10^7 ^cells. The cells were incubated again on ice for 15 minutes, 10 times the labelling volume of depletion buffer was added, and the cells were pelleted by centrifugation at 300 *g *for 10 minutes. Then, the cell pellet was resuspended into 500 μl of depletion buffer, and the cells were separated using the same protocol as for CD14-labelled cells. The CD3-depleted population of PBMCs was shown by flow cytometry to be less than 0.1% CD3^+ ^when stained with anti-CD3 human fluorescein isothiocyanate (BD Biosciences, San Jose, CA, USA).

After macrophage or T-cell depletion in OA synovial cells, a cell count was performed on the retained total population and the depleted population. After the cell count, the two populations were resuspended in RPMI 1640 (with 2 mM l-glutamine and 10 μg/ml penicillin-streptomycin and supplemented with 10% heat-inactivated FCS) at equal cell concentrations and plated at 1 ml/well into a 12-well plate. Due to individual variations between patients, the densities at which cells could be plated varied between 1 × 10^5 ^and 1 × 10^6 ^cells per well, although the total cell population and the depleted cell population were always plated at the same density for each patient. Cells were left to adhere for 24 hours before the supernatants were removed and the cells collected by trypsination and pelleted by centrifugation at 3,000 rpm for 5 minutes.

### Anti-cytokine experiments

In these experiments, OA cultures of synovial cells were prepared using the protocol above. Two million cells were plated into 4 wells on a 24-well plate in 1 ml of RPMI 1640 supplemented with 10% FCS (as above). The cells were left untreated, incubated with the p75 TNF-soluble receptor-immunoglobulin (Ig) fusion protein etanercept (Enbrel; 100 μg/ml), incubated with a neutralising anti-IL-1β antibody purchased from R&D Systems, Inc. (Minneapolis, MN, USA) (10 μg/ml), or incubated with a combination of Enbrel and anti-IL-1β. Although Enbrel binds both TNF-α and lymphotoxin, it would have a selective effect on TNF-α in this system because neither OA nor RA cultures of synovial cells produce detectable amounts of lymphotoxin [28,29]. After incubation for 48 hours at 37°C and 5% CO_2_, the supernatants were removed and the cells collected by trypsination and pelleted by centrifugation at 3,000 rpm for 5 minutes. Higher doses (up to 2 mg/ml Enbrel and up to 200 μg/ml anti-IL-1β) and longer times of exposure (up to 96 hours) of these cytokine inhibitors did not increase the inhibitory effects observed (data not shown). As judged by microscopy, the 3-(4,5-dimethylthiazol-2-yl)-2,5-diphenyl tetrazolium bromide assay, the mRNA levels of the housekeeping gene *GAPDH *(glyceraldehyde-3-phosphate dehydrogenase) (see Figure 5 later in text), and the caspase-3 assay (data not shown), neither of these two cytokine inhibitors caused significant apoptosis or cell death.

### Analysis of cytokines and matrix MMPs

To examine the differences in cytokine production between the primary OA synovial cell cultures and those depleted of macrophages, as well as the synovial cell cultures incubated with anti-cytokine antibodies, the supernatants were examined by enzyme-linked immunosorbent assay (ELISA). Supernatants were analysed for TNF-α, IL-1β, IL-6, IL-8, and monocyte chemoattractant protein-1 (MCP-1) by ELISA kits purchased from Biosource International (Camarillo, CA, USA) and R&D Systems, Inc. The production of MMP-1, MMP-3, MMP-9, MMP-13, and their inhibitor TIMP-1 was also examined by ELISA as described [28].

### RNA extraction and reverse transcription-polymerase chain reaction analysis

Total RNA was isolated from cell pellets as previously described [30] and isolated using TRIreagent (Sigma-Aldrich) and RNeasy (Qiagen Ltd., Crawley, UK) according to the manufacturers' protocols. Reverse transcription-polymerase chain reaction (RT-PCR) analysis was performed using an RNA PCR kit (PerkinElmer LAS (UK) Ltd, Beaconsfield, Bucks, UK) as described [31] using oligonucleotide primers corresponding to cDNA sequences for the ADAMTS4 and ADAMTS5 aggrecanases, MMPs, TIMPs, and link protein (Table 1). After an initial denaturation step of 1 minute at 95°C, amplification consisted of between 30 and 60 cycles of 1 minute at 95°C, 45 seconds at the primer annealing temperature, 30 seconds at 72°C followed by a final extension step of 5 minutes at 72°C. The PCR products were visualised on a 2% agarose gel (containing 0.5 μg/ml ethidium bromide), and their nucleotide sequences were verified using an ABI 310 Genetic Analyzer (Applied Biosystems, Foster City, CA, USA). The NIH Imaging system (National Institutes of Health, Bethesda, MD, USA) was used to quantify the bands on the gels and to standardise them against the GAPDH control.

## Results

OA synovial cell cultures spontaneously secrete a variety of pro- and anti-inflammatory cytokines in quantities readily detectable by ELISA [28]. These mediators include the macrophage-produced TNF-α, IL-1β, and large amounts of IL-6, IL-8, and MCP-1. They also produce large amounts of the major MMPs (1, 3, 9, and 13) and their main endogenous inhibitor, TIMP-1 (Table 2). Importantly, it is also possible to study the expression of various degradative enzymes by RT-PCR.

### Macrophage and T-cell depletion studies

**Figure 1:**
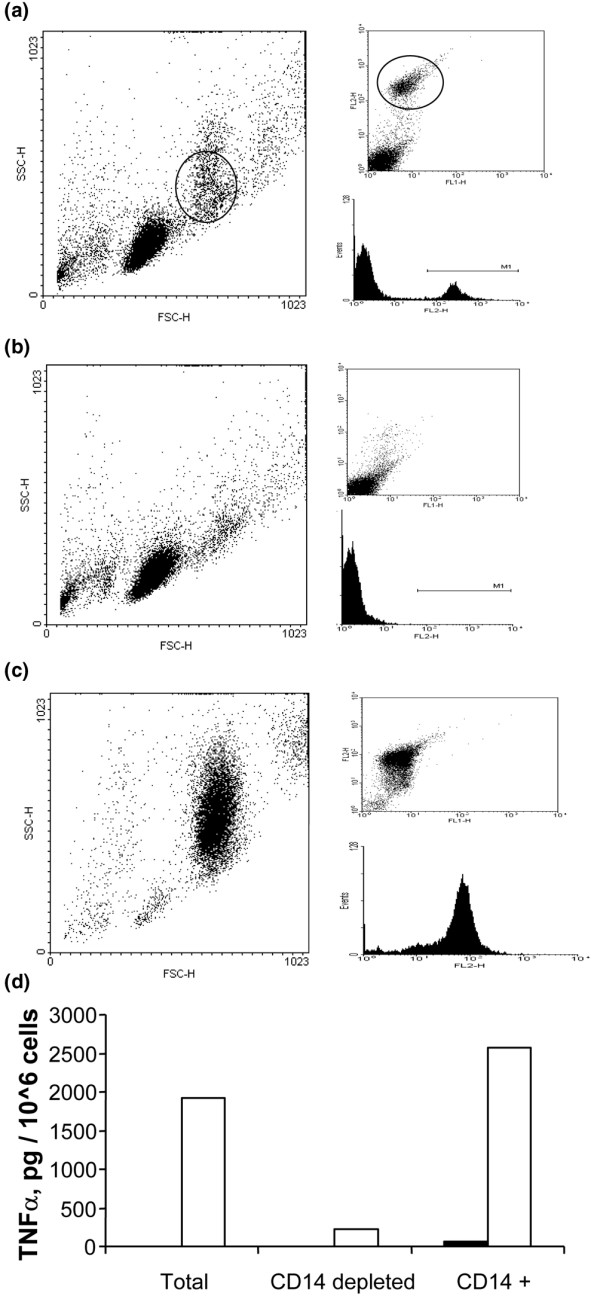
Fluorescence-activated cell sorting (FACS) analysis of monocyte/macrophage depletion in peripheral blood mononuclear cells (PBMC). **(a) **The left panel shows FACS analysis of forward scatter (FSC) and side scatter (SSC) of unlabeled PBMC, with the approximate monocyte population indicated with a circle. In the right panels, cells have been incubated with an anti-CD14 phycoerythrin antibody, clearly showing the distinct population of CD14^+ ^cells on FACS (upper panel) and in a histogram (lower panel). **(b) **The corresponding panels show monocyte-depleted PBMC. In the left panel, the monocyte population is reduced, although the debris and cell clusters blur this effect. In the right upper panel, the CD14^+ ^population virtually disappears, as confirmed by the histogram in the right lower panel. **(c) **The corresponding panels show the enriched monocytes/macrophages. In the left panel, although there is still a fair amount of debris and cell clusters, the monocyte population is considerably enriched. In the right upper panel, the CD14^+ ^population is also very much enriched, as confirmed by the histogram in the panel below. **(d) **A functional assay using enzyme-linked immunosorbent assay analysis of spontaneous and lipopolysaccharide-stimulated (open bars) tumour necrosis factor-alpha (TNF-α) production in monocyte/macrophage-depleted and -undepleted PBMCs verifies that the monocyte/macrophage depletion is effective.

A method of isolating monocytes/macrophages or T cells by using separator columns and anti-CD14 or anti-CD3 antibodies coupled with magnetic beads was first set up in peripheral blood by using antibodies purchased from Miltenyi Biotec. As judged by fluorescence-activated cell sorting (FACS) analysis using fluorescent antibodies to detect the monocyte and lymphocyte populations and by functional studies (lipopolysaccharide-induced TNF-α production), this method of monocyte depletion worked well in the PBMC model (Figure 1).

**Figure 2:**
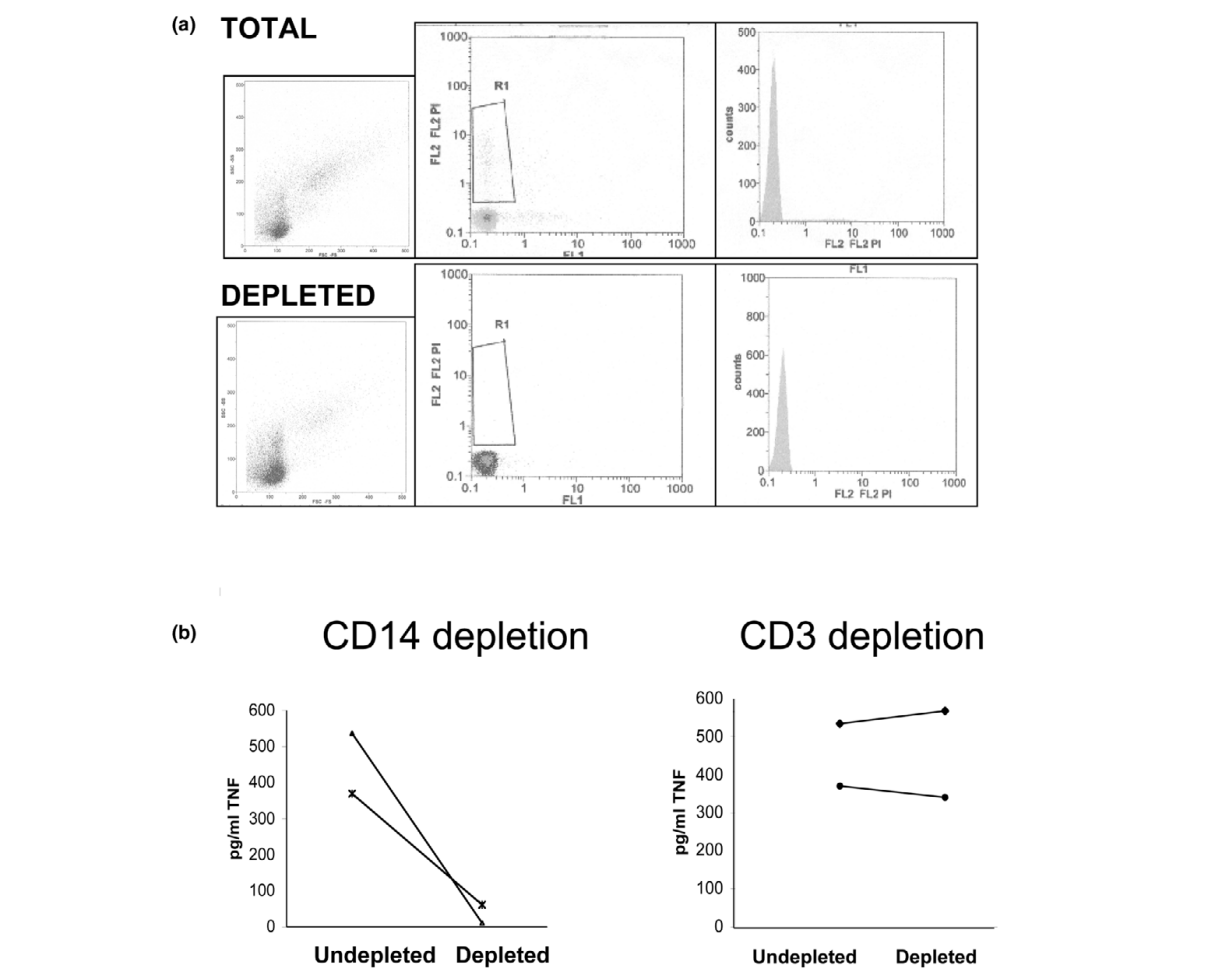
FACS analysis of OA synovial cells. **(a) **Fluorescence-activated cell sorting (FACS) analysis of total (upper three panels) and CD14^+^-depleted (lower three panels) osteoarthritis (OA) cultures of synovial cells. The upper three panels show FACS analysis of forward scatter/side scatter (FSC/SSC) (left panel), analysis of FL2^+ ^cells after incubation with the anti-CD14 phycoerythrin antibody with the CD14^+ ^population indicated in region R2 (middle panel), and the CD14^+ ^cells in this region showing as FL2^+ ^in a histogram (right panel). The corresponding lower three panels show diminution of the macrophage population on FSC/SSC (left panel), disappearance of CD14^+ ^cells (middle panel), and a histogram (right panel) after depletion of CD14^+ ^cells. **(b) **A functional assay using the spontaneous tumour necrosis factor-alpha (TNF-α) production from the OA synovial cells shows effective inhibition of TNF-α production in CD14^+^-depleted cells from two patients (left panel), whereas CD3^+ ^cell depletion has no effect.

The same method was then used in OA cultures of synovial cells. FACS analysis again demonstrated effective depletion of macrophages from the synovial cell cultures and recovery of a relatively pure macrophage population from the column (Figure 2a). There was a marked reduction in secreted TNF-α from macrophage-depleted cultures (CD14), indicating effective depletion, whereas T-cell depletion (CD3) had no effect (Figure 2b), thus ruling out significant non-specific binding to the column. Due to the extremely low number of T cells in the OA synovium (<0.1%), it was not possible to detect the OA synovial T-cell population by FACS analysis.

**Figure 3:**
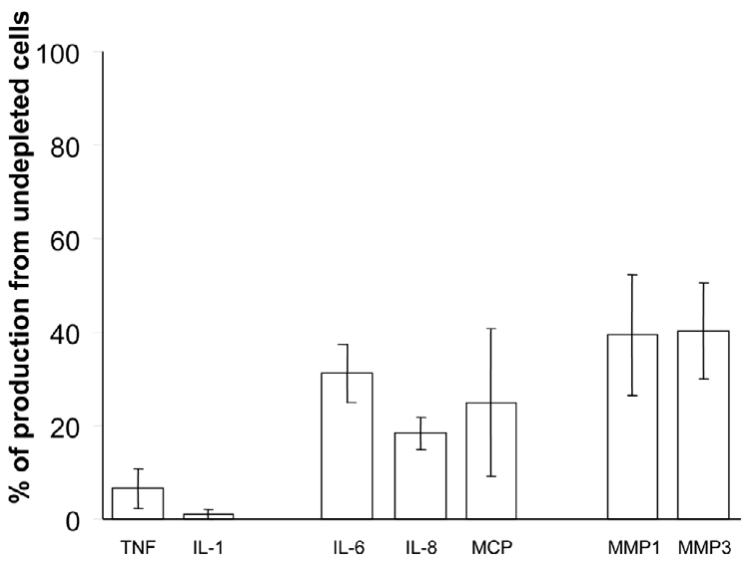
Effect of macrophage depletion on cytokine and matrix metalloproteinase (MMP) production in osteoarthritis (OA) synovial cells. OA cultures of synovial cells were either left intact or macrophage-depleted, as described in Materials and methods. Cells were left to adhere for 24 hours before the supernatants were removed for enzyme-linked immunosorbent assay analysis of cytokines and MMPs. Data are expressed as the percentage of cytokine/MMP production in the depleted culture as compared with the undepleted one. The standard error of the mean is given (*n *= 7–9). IL, interleukin; MCP, monocyte chemoattractant protein; TNF, tumour necrosis factor.

In a series of 10 experiments using samples from different patients, OA synovium was digested as specified above. Half of the sample was put into culture directly and the other half was macrophage-depleted before being put to culture. The spontaneous production of TNF-α and IL-1 was almost totally inhibited in the CD14^+^-depleted cultures (Figure 3), which is as it should be because these two cytokines are macrophage-produced. Interestingly, there was also potent (70% to 80%) inhibition of several proinflammatory cytokines (namely IL-6, IL-8, and MCP-1) produced mainly by synovial fibroblasts. Both MMP-1 and MMP-3 were also decreased by 60%, suggesting that the macrophage is a major regulator of the production of both MMPs and proinflammatory cytokines (Figure 3).

### Inhibition of macrophage-produced cytokines

**Figure 4:**
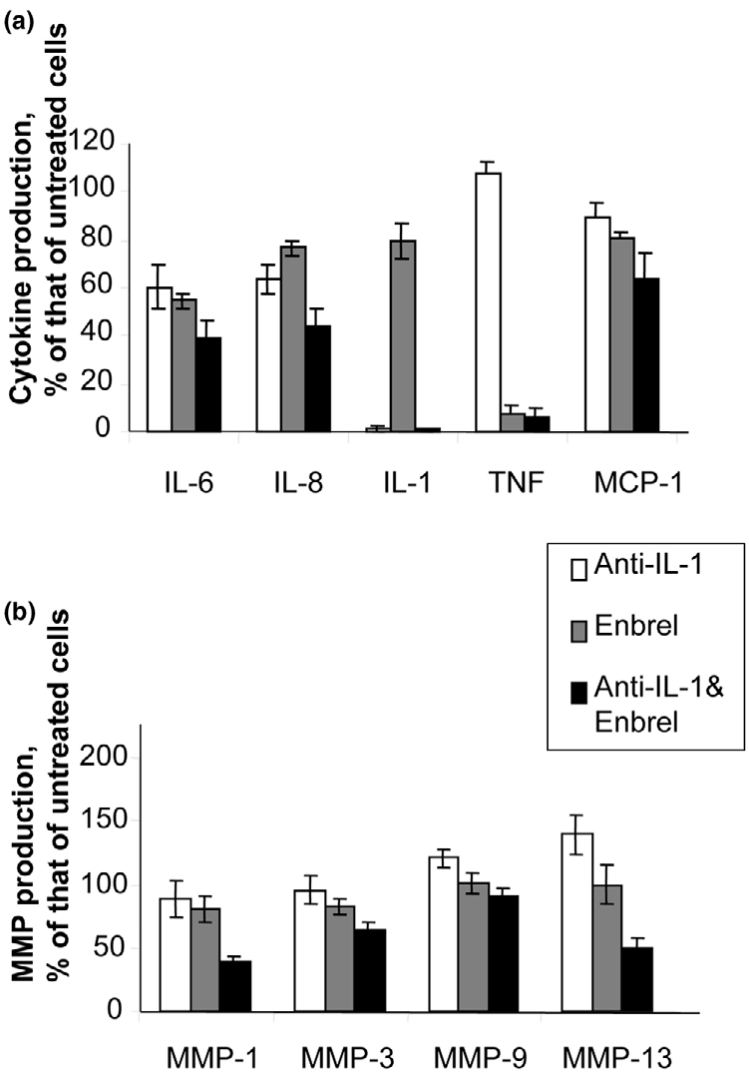
Effect of neutralisation of tumour necrosis factor (TNF)-α and/or interleukin (IL)-1 on cytokine and matrix metalloproteinase (MMP) production in osteoarthritis synovial cells. In these experiments, 2 × 10^6 ^cells per well were plated into 4 wells on a 24-well plate in 1 ml of RPMI 1640 supplemented with 10% foetal calf serum. The cells in these 4 wells were left untreated, incubated with the p75 TNF-soluble receptor-immunoglobulin fusion protein etanercept (Enbrel), incubated with a neutralising anti-IL-1β antibody, or incubated with a combination of etanercept and anti-IL-1β, as described in Materials and methods. After incubation for 48 hours, the supernatants were removed for enzyme-linked immunosorbent assay analysis of various cytokines **(a) **and MMPs **(b)**. The data are expressed as percentage of the production of untreated cells, and the standard error of the mean is given (*n *= 6–7).

A series of nine experiments were performed using OA synovium from different donors, digested and cultured as specified above, to investigate the effect of selective inhibition of TNF-α (via excess of the soluble TNF receptor-Ig fusion protein Enbrel) and/or IL-1 (via excess of a neutralising anti-IL-1β antibody) on various cytokines and MMPs. These OA cultures of synovial cells were left untreated or were treated with excess neutralising anti-IL-1β antibody, excess Enbrel, or both of these anti-cytokine strategies. There was effective neutralisation of TNF-α and IL-1β in cultures treated with Enbrel and the neutralising anti-IL-1β antibody, respectively (Figure 4a). Importantly, we could observe no significant inhibitory effect of Enbrel on IL-1β production or of the neutralising anti-IL-1β antibody on TNF-α production (Figure 4a), irrespective of dose of the cytokine inhibitor in question and of time of exposure. This is in contrast to the situation in RA, in which IL-1 is strongly TNF-dependent in cultures of synovial cells [32]; these results would indicate that in OA, there is a redundancy between IL-1 and TNF, rather than a 'cytokine cascade' with either of these cytokines controlling the production of the other (Figure 4a). Both Enbrel and the neutralising anti-IL-1β antibody decreased the spontaneous production of IL-6 by 40% (*p *< 0.01); there was an additive effect, and 60% inhibition (*p *< 0.001) was achieved when both IL-1 and TNF were neutralised. The production of IL-8 was decreased by either Enbrel (*p *< 0.05) or the neutralising anti-IL-1β antibody (*p *< 0.01); again there was an additive effect, and 60% inhibition (*p *< 0.001) was achieved when both IL-1 and TNF were neutralised. The production of MCP-1 was not significantly affected by the neutralising anti-IL-1β antibody, although it was decreased (*p *< 0.05) by Enbrel or the combination of the two (*p *< 0.01) (Figure 4a).

We also studied the effect of Enbrel and/or the neutralising anti-IL-1β antibody on the spontaneous production of the major MMPs (1, 3, 9, and 13). The neutralising anti-IL-1β antibody alone had no significant effect on any of these MMPs (Figure 4b). Enbrel induced a significant (*p *< 0.05) reduction of MMP-1 and MMP-3 production. In contrast, all of these MMPs were significantly inhibited by the combination of Enbrel and the neutralising anti-IL-1β antibody. The production of the important collagenases MMP-1 and MMP-13 was potently decreased (50% to 60%; *p *< 0.001), and the production of MMP-3 (*p *< 0.01) and MMP-9 (*p *< 0.05) was also downregulated. There was no effect of either anti-cytokine strategy on the spontaneous production of TIMP-1 (data not shown).

### Analysis of RNA expression for MMPs, TIMPs, and aggrecanases using RT-PCR

**Figure 5:**
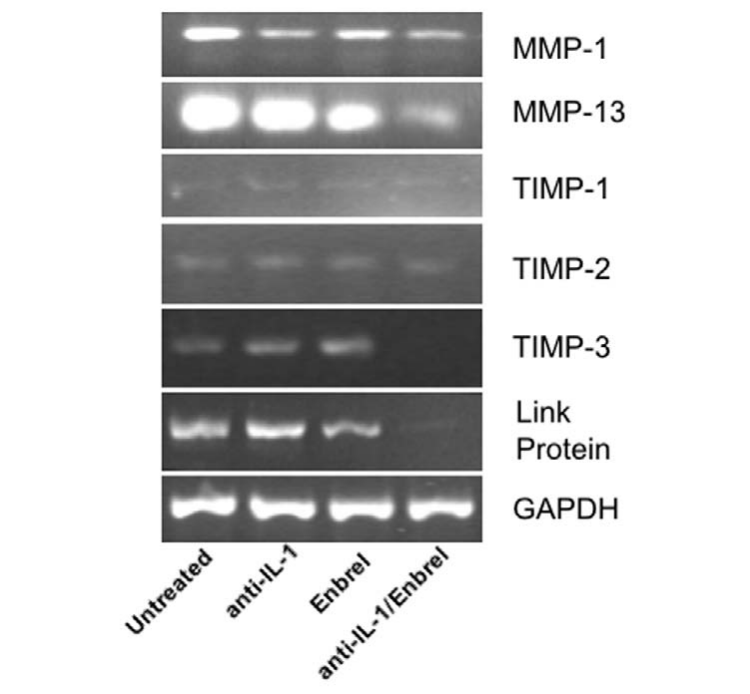
Effect of neutralisation of tumour necrosis factor (TNF)-α and/or interleukin (IL)-1 on the expression of matrix metalloproteinases (MMPs), tissue inhibitors of MMP (TIMPs), and link protein in osteoarthritis synovial cell cultures. Two million cells per well were plated into 4 wells on a 24-well plate and left untreated, incubated with the p75 TNF-soluble receptor-immunoglobulin fusion protein etanercept (Enbrel), incubated with a neutralising anti-IL-1β antibody, or incubated with a combination of etanercept and anti-IL-1β, as described in Materials and methods. After incubation for 48 hours, the cells were washed with phosphate-buffered saline and the RNA extracted using Tri-reagent for reverse transcription-polymerase chain reaction analysis with oligonucleotide primers specific for MMP-1, MMP-3, MMP-13, TIMP-1, TIMP-2, TIMP-3, and link protein. In all panels, analysis of GAPDH (glyceraldehyde-3-phosphate dehydrogenase) was used for comparison of gene expression.

**Figure 6:**
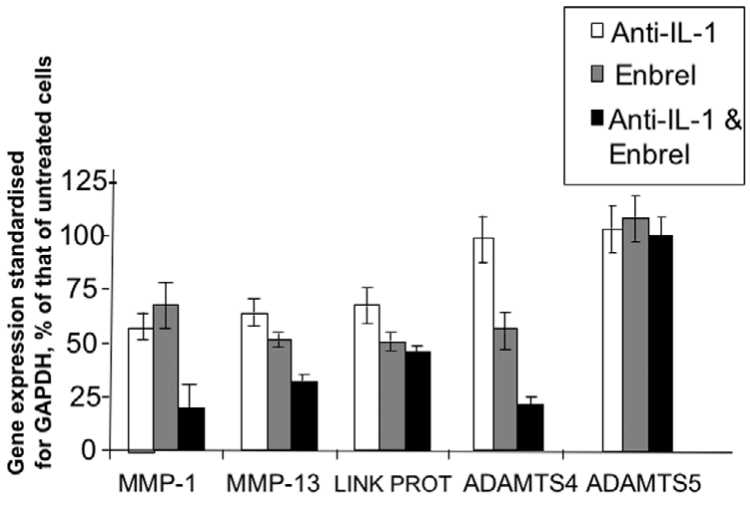
Polymerase chain reaction data from matrix metalloproteinase (MMP), link protein, and ADAMTS analysis, derived as described in Materials and methods and in Figures 5 and 7. Data are expressed as percentage of the gene expression in untreated cells, as standardised for GAPDH (glyceraldehyde-3-phosphate dehydrogenase). The standard error of the mean is given (*n *= 4–5). ADAMTS, a disintegrin and metalloprotease with thrombospondin motifs; IL, interleukin.

Using RT-PCR technology, we investigated the effect of selective inhibition of TNF-α and/or IL-1 on MMPs, TIMPs, and ADAMTS metalloproteinases in the OA synovial cell cultures. These experiments used the cell pellets from the experiments described earlier (eight patients in all), which were freezer-stored before RNA was extracted and analysed using RT-PCR. MMP-1 was significantly (*p *< 0.05) inhibited by neutralising IL-1 but only marginally affected by Enbrel. Neutralising both cytokines led to significant (*p *< 0.01) inhibition of MMP-1 (Figures 5 and 6). With regard to MMP-13, the effect of neutralising each cytokine on its own was again less potent, although a combination of Enbrel and the neutralising anti-IL-1β antibody led to significant (*p *< 0.01) downregulation of this enzyme via a synergistic effect (Figures 5 and 6). The effect on MMP-3 was similar (data not shown). With regard to the expression of various TIMPs, we observed that although TIMP-1 and TIMP-2 were unaffected, TIMP-3 was inhibited by a combination of Enbrel and the neutralising anti-IL-1β antibody (Figure 5). These PCR results agree well with the ELISA results described earlier (Figure 4b). The reason the effects observed are more marked in the PCR system is probably that when the OA synovial cells are put into culture, they are highly active and produce large amounts of MMPs. With time, this is inhibited by neutralisation of the macrophage-produced, proinflammatory cytokines driving MMP production, but by then, large quantities of these MMPs have already been produced, thus blurring the results. In contrast, the PCR findings reflect the state of the cells 2 days after addition of the Enbrel and/or the neutralising anti-IL-1β antibody. There appears to be a discrepancy with regard to MMP-1: whereas the neutralising anti-IL-1β antibody decreases its expression at the RNA level (Figure 5), there is no significant effect on its production (Figure 4b). This might be explained by the activation of latent MMP activity but no further production of 'new' enzyme.

Surprisingly, we also found that link protein is significantly (*p *< 0.01) inhibited by Enbrel or by a combination of Enbrel and the neutralising anti-IL-1β antibody (Figures 5 and 6). These results suggest that anabolic synthesis of aggrecan aggregate components may be affected by neutralisation of these cytokines.

**Figure 7:**
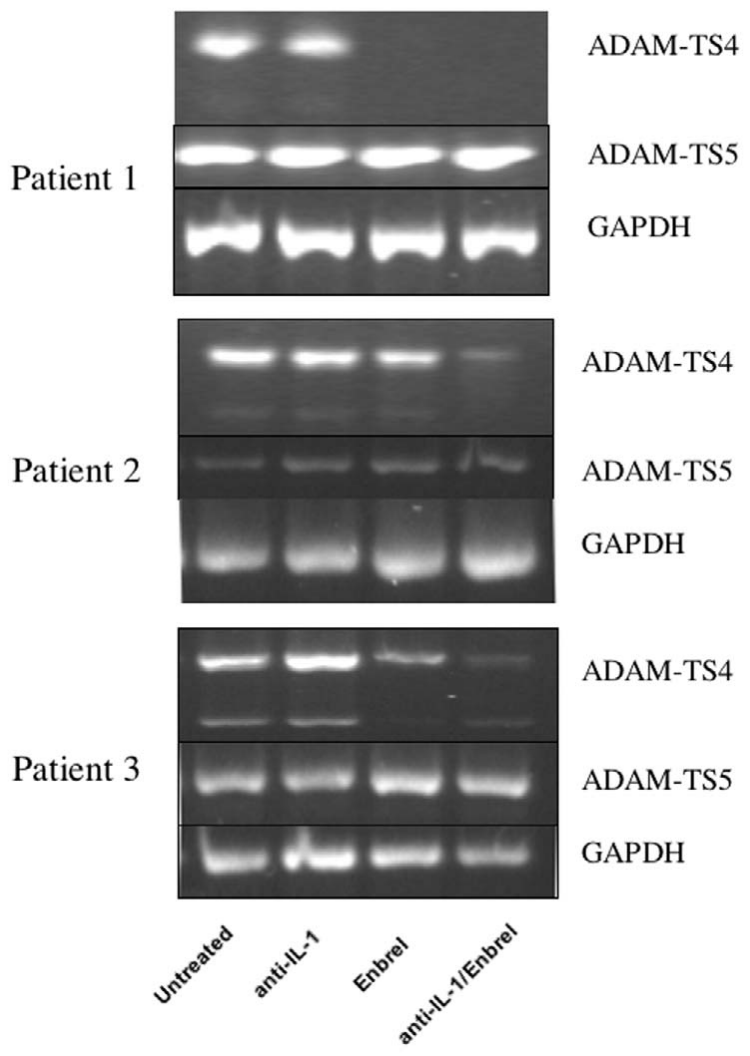
Effect of neutralisation of tumour necrosis factor (TNF)-α and/or interleukin (IL)-1 on the expression of the ADAMTS4 and ADAMTS5 aggrecanases in osteoarthritis synovial cell cultures. Two million cells per well were plated into 4 wells on a 24-well plate and left untreated, incubated with the p75 TNF-soluble receptor-immunoglobulin fusion protein etanercept (Enbrel), incubated with a neutralising anti-IL-1β antibody, or incubated with a combination of etanercept and anti-IL-1β, as described in Materials and methods. After incubation for 48 hours, the cells were washed with phosphate-buffered saline and the RNA extracted using Tri-reagent for reverse transcription-polymerase chain reaction analysis with oligonucleotide primers specific for ADAMTS4 and ADAMTS5 in three patients. In all panels, analysis of GAPDH (glyceraldehyde-3-phosphate dehydrogenase) was used for comparison of gene expression. ADAMTS, a disintegrin and metalloprotease with thrombospondin motifs.

We also studied the expression of the aggrecanases ADAMTS4 and ADAMTS5 in five patients. There was no effect of either Enbrel or the neutralising anti-IL-1β antibody on ADAMTS5 expression, nor was it at all affected by a combination of these treatments (Figure 7). Thus, this aggrecanase appears to be constitutive in OA cultures of synovial cells. In contrast, ADAMTS4 was significantly (*p *< 0.05) inhibited by Enbrel and more potently (*p *< 0.01) inhibited by a combination of Enbrel and the neutralising anti-IL-1β antibody (Figures 6 and 7). The lower band in the ADAMTS4 panel in Figure 7 represents a newly discovered splice variant of ADAMTS4 [33].

## Discussion

In recent years, its pivotal importance in RA having been clearly established, the synovial macrophage has begun to attract interest in OA also. Imaging studies have demonstrated synovitis in both early and late OA [34-36], even in joints in which it could not be detected clinically. Immunohistochemical studies have found that, particularly in early OA, this synovitis has a mononuclear cell infiltrate, with considerable production of proinflammatory cytokines like TNF-α and IL-1β and destructive enzymes like MMP-1 and MMP-3 [2,37]. This agrees well with our data from the OA synovial cell culture model, which exhibits spontaneous production of TNF-α and IL-1β and many other cytokines and MMPs.

The first major finding from this study is that OA cultures of synovial cells can be selectively depleted of macrophages and that macrophage depletion results in downregulation of several fibroblast-produced cytokines and MMPs (Figures 2 and 3). This would indicate that in these densely plated cultures, the macrophages play a role in perpetuating inflammatory and destructive responses from the synovial fibroblasts. The cultures enriched for OA macrophages produce only very limited amounts of these MMPs (data not shown), and thus the macrophage depletion *per se *cannot be held responsible for this effect. That the regulation is not tighter than observed is probably due to the fibroblasts having quite an active phenotype already when taken into culture, with considerable spontaneous production of various mediators. But once the macrophages are removed, the synovial fibroblasts downregulate their production of both proinflammatory cytokines and destructive MMPs.

In a mouse model of experimental OA, synovial lining macrophages are pivotal cells, mediating osteophyte formation and other OA-related pathology [15]. The effects of macrophage depletion observed in the present study may well provide an explanation of these effects, either directly due to the inhibition of fibroblast-produced cytokines and/or MMPs or indirectly due to cytokine-mediated inhibition of the production of growth factors [16].

The majority of studies of the regulation of cytokines and destructive enzymes in arthritis have relied on the stimulation of outgrown synovial fibroblasts with various catalytic molecules, among them TNF-α and IL-1β. To investigate the mechanisms behind the macrophage stimulation of other cells in OA cultures of synovial cells, we instead used specific neutralisation of TNF-α and/or IL-1β, as previously described in RA [32,38]. The second major finding from this study is that although the effects of inhibiting each cytokine on its own are less impressive, neutralisation of both TNF-α and IL-1β results in significant inhibition of fibroblast-produced cytokines and MMPs to a degree roughly comparable to that induced by macrophage depletion. This would suggest that in these OA cultures of synovial cells, the macrophage perpetuates inflammatory and destructive responses from the synovial fibroblasts through a combination of TNF-α and IL-1β (Figures 4 and 5). There appears to be a redundancy in the system, and the neutralisation of just one of these cytokines is not sufficient. The reason the effect of macrophage depletion, or combined inhibition of TNF-α and IL-1β, does not inhibit IL-6 and IL-8 by more than 60% is probably due to the fact that the OA synovial fibroblasts express an activated phenotype when put into culture and are thus able to produce both cytokines and MMPs for some time before being gradually downregulated when the stimulus is removed.

In RA, it is well established that there is a 'cytokine cascade' with TNF-α at the top. The production of IL-1, IL-6, IL-8, and granulocyte macrophage colony-stimulating factor is inhibited when TNF-α is neutralised [32], a discovery that has led to the emergence of anti-TNF-α therapy in RA [39]. It is known that the macrophage signal transduction leading to TNF-α and IL-1β induction is stimulus-specific with regard to nuclear factor-kappa B (NF-κB) dependence [40-42], and some data support differences in the regulation of TNF-α and IL-1β in RA and in OA, with stronger NF-κB dependence observed in RA [[38,43,44] versus [28]]. The third major finding in this study points out another difference between the RA and the OA synovium: IL-1β is not TNF-α-dependent. Nor is there any effect on TNF-α production when IL-1β is neutralised, again suggesting a redundancy between these two cytokines in the OA synovium (Figure 4a). The possibility of using anti-IL-1 or anti-TNF-α strategies in OA has been discussed for some time, with some early reports on record [45,46]. Due to the absence of a 'cytokine cascade' in OA with regard to the regulation of TNF-α and IL-1β in the OA synovium (as demonstrated here), it appears unlikely that the neutralisation of either of these cytokines would affect the production of the other. It is still quite possible that either TNF-α or IL-1β has other significant downstream effects to render such therapy worthwhile, particularly with regard to synovial inflammation.

The fourth major finding from this study is that, whereas ADAMTS5 is not driven by TNF-α or IL-1β in the OA synovium, ADAMTS4 expression is reduced by TNF-α blockade and is significantly downregulated when both TNF-α and IL-1β are neutralised (Figures 6 and 7). This finding will have significance for further research concerning the regulation of these enzymes. There is evidence that ADAMTS4 is upregulated by IL-1 in various cartilage systems [27,47,48], but this is the first study to demonstrate that in the OA synovium, this aggrecanase is driven by TNF-α and IL-1β. In another study, using human OA fibroblasts, we found that ADAMTS4, but not ADAMTS5, could be induced with either IL-1 or TNF-α in an NF-κB-dependent manner [49]. Although studies using transgenic mice [50-52] suggest that in these murine models of degenerative joint disease, ADAMTS5 is the pathologically induced aggrecanase, our data suggest that ADAMTS4 is the aggrecanase induced by proinflammatory cytokines in the human OA synovium. The identification of the primary aggrecanase (ADAMTS4 or ADAMTS5) involved in human OA still needs to be conclusively established.

## Conclusion

The results discussed above, as well as data from other groups, would support the view that the macrophage is an important player in promoting the production of inflammatory and degradative mediators in the OA synovium. In particular, our results indicate a role for both TNF-α and IL-1β in driving inflammatory responses in OA. This would give priority to attempts to modify macrophage function in OA, with the aim of decreasing both inflammatory synovitis and the production of degradative enzymes of importance for the progression of the disease. Such studies would be of clear importance for drug discovery and for the determination of novel therapeutic targets in OA.

## Abbreviations

ADAMTS = a disintegrin and metalloprotease with thrombospondin motifs; ELISA = enzyme-linked immunosorbent assay; FACS = fluorescence-activated cell sorting; FCS = foetal calf serum; GAPDH = glyceraldehyde-3-phosphate dehydrogenase; Ig = immunoglobulin; IL = interleukin; MACS = magnetic-activated cell sorting; MCP-1 = monocyte chemoattractant protein-1; MMP = matrix metalloproteinase; NF-κB = nuclear factor-kappa B; OA = osteoarthritis; PBMC = peripheral blood mononuclear cell; RA = rheumatoid arthritis; RT-PCR = reverse transcription-polymerase chain reaction; TIMP = tissue inhibitor of matrix metalloproteinase; TNF-α = tumour necrosis factor-alpha.

## Competing interests

The authors declare that they have no competing interests.

## Authors' contributions

JB designed the study, performed experiments throughout, and wrote the manuscript. SDW designed primers and performed many PCR experiments. SL and NA performed most of the depletion, cytokine inhibition, and ELISA experiments. CEH provided input on study design and PCR experiments. All authors read and approved the final manuscript.

## Figures and Tables

**Table 1:** Oligonucleotide primers used for reverse transcription-polymerase chain reaction

Target template	Polymerase chain reaction primers	Product size (bp)	Annealing temperature (°C)
GAPDH	5'-TGGTATCGTGGAAGGACTCAT 5'-GTGGGTGTCGCTGTTGAAGTC	370	53
ADAMTS4	5'-GTCTGTGTCCAGGGCCGATGC 5'-GCCGCCGAAGGATCTCCAGAA	541	61.8
ADAMTS5	5'-GCGGATGTGTGCAAGCTGACC 5'-AGTAGCCCATGCCATGCAGGA	487	57.4
MMP-1	5'-ACAAATCCCTTCTACCCGGAA 5'-GGATCCATAGATCGTTTATAT	314	50.8
MMP-3	5'-CTTTTGGCGAAAATCTCTCAG 5'-AAAGAAACCCAAATGCTTCAA	404	50
MMP-9	5'-GGCCTCCAACCACCACCACA 5'-CGCCCAGAGAAGAAGAAAAGC	400	61
MMP-13	5'-TTCTGGCACACGCTTTTCCTC 5'-GGTTGGGGTCTTCATCTCCTG	273	53
TIMP-1	5'-CCACCTTATACCAGCGTTAT 5'-CCTCACAGCCAACAGTGTAGG	282	54
TIMP-2	5'-GTGGACTCTGGAAACGACAT 5'-TCTTCTTCTGGGTGGTGCTCA	265	54
TIMP-3	5'-GGGAAGAAGCTGGTAAAGGAG 5'-GCCGGATGCAGGCGTAGTGTT	418	54
Link protein	5'-GGAAGCAGAGCAAGCCAAGGT 5'-GAAGGAGGCGATCACAGCATC	448	56.1

Primer sequences correspond to sequences for human cDNAs deposited to GenBank. Where a mixed base is indicated (that is, for GAPDH), the sequence also corresponds to the analogous rat cDNA. ADAMTS, a disintegrin and metalloprotease with thrombospondin motifs; GAPDH, glyceraldehyde-3-phosphate dehydrogenase; MMP, matrix metalloproteinase; TIMP, tissue inhibitor of matrix metalloproteinase.

**Table 2:** Production of cytokines and matrix metalloproteinases

	Untreated		Anti-IL-1		Enbrel		Anti-IL-1 and Enbrel	
	
	Mean	SEM	Mean	SEM	Mean	SEM	Mean	SEM
TNF (*n *= 7)	14.035	5.208	10.863	3.524	1.262	0.4787	1.291	0.5701
IL-1 (*n *= 7)	2.0587	1.216	0.00357	0.003858	0.767	0.310	0.002857	0.00291
IL-6 (*n *= 7)	1,218,830	374,998	735,202.7	180398.9	725,517	187,147.4	378,930.3	103,236.2
IL-8 (*n *= 7)	325.62	108.67	211.81	64.35	245.04	75.69	134.75	37.48
MCP-1 (*n *= 7)	62.29	15.43	60.29	16.17	51.58	13.95	41.56	11.65
MMP-1 (*n *= 7)	1,398.1	513.0	1,431.9	617.6	1,083.4	448.9	644.9	242.7
MMP-3 (*n *= 7)	4,508.7	1,840.7	4,920.8	2,197.1	3,953.4	1,789.2	3,169.7	1,543.6
MMP-9 (*n *= 6)	23.5	7.2	23.67	6.589	20.83	6.37	19.83	6.427
MMP-13 (*n *= 7)	3,772.3	1,428.5	4,262.7	1,312.1	2,730.5	715.4	1,644	538.2

IL, interleukin; MCP, monocyte chemoattractant protein; MMP, matrix metalloproteinase; SEM, standard error of the mean; TNF, tumour necrosis factor.
